# Supplementary material for: A macroscopic object passively cooled into its quantum ground state of motion beyond single-mode cooling
Source: Nat Commun. 2021 Oct 26;12:6182. doi: 10.1038/s41467-021-26457-8 (PMC8548555; doi:10.1038/s41467-021-26457-8)
Supplement: Supplementary file 1 — Supplementary Information [file 41467_2021_26457_MOESM1_ESM.pdf]

SUPPLEMENTARY INFORMATION for A macroscopic object passively cooled into its quantum ground state of motion beyond single-mode cooling

Supplementary Note 1: Setup

A. Microwave wiring

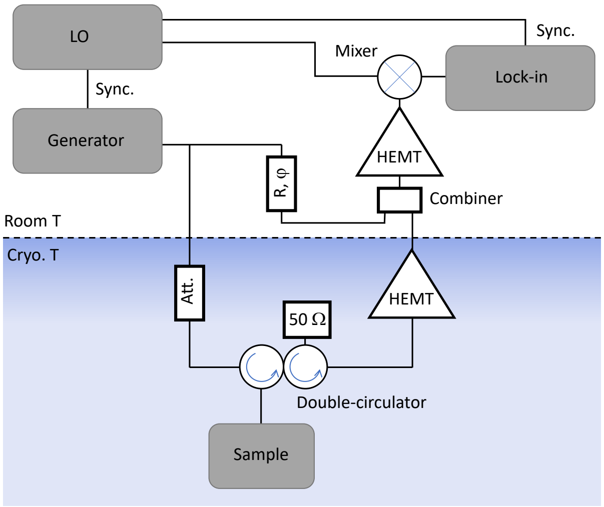

Supplementary Figure 1: **Microwave circuit schematic.** Simplified circuitry of the microwave setup (note attenuation, circulators with 50 Ω load, $R-\phi$ opposition settings); see Ref. [33] for details.

The microwave setup is a completely conventional one. Two platforms have been equipped and calibrated: a commercial dry Bluefors® cryostat for preliminary measurements (not presented in the core of the manuscript), and the home-made wet nuclear demagnetization cryostat. On the Bluefors®, a LNF® cryo-HEMT is present on the 4 K stage with a power combiner mounted in front of it allowing to cancel the strong pump tone (avoiding amplifier saturation). On the nuclear demag. it is a Caltech® cryo-HEMT bolted on the 4 K stage. Both have a second room temperature LNF® HEMT amplifier. On the nuclear demag. cryostat the "opposition line" made with the power combiner is at room temperature; the Caltech amplifier is linear enough so that it never saturates with the pump powers we use (from an Agilent® 20 GHz synthesiser). After mixing up the signal with a local tone (LO) shifted by $\pm\Omega_m + 2$ MHz, the measurement is performed with a Zurich Instrument® lock-in that is demodulated at the shift frequency (used in spectrum mode). A basic drawing is provided in Suppl. Fig. 1. The coaxial lines from room temperature to 4 K are stainless steel, and all the cryogenic routing below is NbTi apart from the mixing chamber level which is copper. Details with complete schematics can be found in Ref. [33] and Ref. [61]. The noise background (brought back at input of the cryogenic HEMT) is on the nuclear demag. setup about 100 photons in this experiment. Note that this is clearly not single-shot, but the circuit has the valuable advantage of being simple.

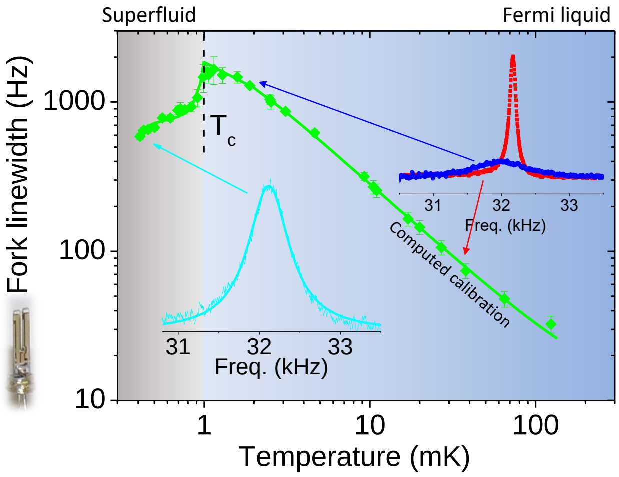

Supplementary Figure 2: **Tuning fork ^{3}He thermometry.** Main: temperature dependence of the damping (in Hz) measured on the tuning fork. Insets: Lorentzian resonance peaks of the tuning fork corresponding to different working points. See Ref. [33] for details. Error bars correspond to type A uncertainties.

B. Cryogenics

The nuclear demagnetization cryostat is made of two stages: a (conventional) home-made dilution unit that reaches base temperature of 10 mK, followed by a copper laminar nuclear stage on which the experimental cell is mounted [62]. The two stages are connected to each other through a Lancaster-made aluminium heat switch [63]. On the dilution unit, the temperature is inferred from a calibrated carbon resistor and a commercial magnetic field fluctuation thermometer (MFFT) from Magnicon®. On the nuclear stage we use a home-made ^{3}He thermometer: a quartz tuning fork immersed in the fluid that measures its viscosity [64]. This (almost) primary thermometry also provides a fixed temperature point: the

superfluid transition T_c at 0.95 mK (for 0 bar pressure). Calibration of the thermometer is shown in Suppl. Fig. 2. Note that near T_c the fork is very strongly damped, and the sensitivity is rather poor; however above and below, we can infer temperature very accurately. Details can be looked at in Ref. [33] and Ref. [61].

The ultra-low temperature cooling process is single shot, and starts with a precooling period of about a week: the heat switch is closed (a small magnetic field is applied on the aluminium heat switch to make it normal), and the copper is cooled to (almost) the mixing chamber temperature while a 7 T field is applied to it. The coil producing this field is well compensated, and the cell is surrounded by a superconducting lead shield. After that, the heat switch is opened (the aluminium is made superconducting by removing the small field in the switch), and the large field of 7 T is slowly decreased down to a final value $B_{\rm fin}$ (always larger than 100 mT); this makes the nuclear spins of the copper atoms cool down, which in turn cool down the conduction electrons. The cryostat can stay cold for about a week at an almost fixed temperature (see e.g. Ref. [65] for details on the technique). The beginning of the cooling process is shown in Fig. [1] top inset of the paper.

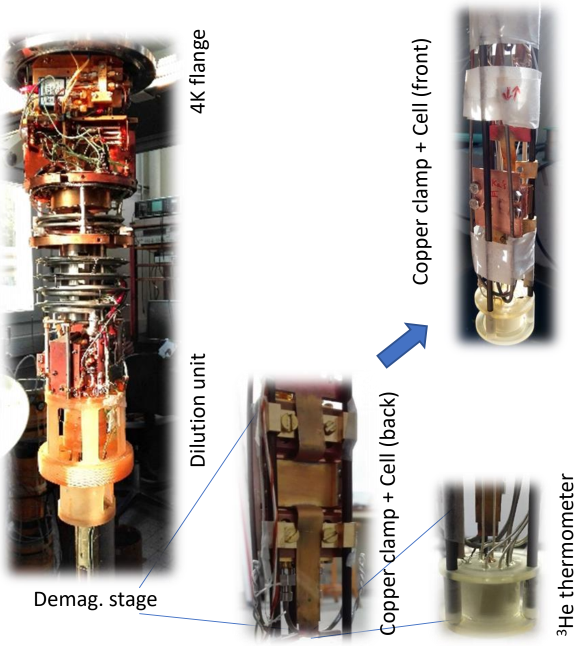

Supplementary Figure 3: **Nuclear demag. cryostat and copper cell.** Left: picture of the dilution unit stage of the nuclear demagnetisation cryostat. Right: bottom of the nuclear stage (outside of the large demag. field zone) with the copper cell mounted (back: "weakly-coupled" drum run, front: this run). The ^{3}He thermometer is also shown.

C. Multiple runs

3 different chips have been measured on the 2 platforms. They are mounted in copper cells with SMA connectors. On the nuclear demag. cryostat, the cell is bolted on a copper clamp that is located at the bottom of the laminar copper stage; the ^{3}He thermometer is just underneath it. The copper clamp is connected to the stage and the thermometer on either side with 1 mm diameter annealed silver wires. Pictures of the demag. cryostat are shown in Suppl. Fig. 3.

Of the 3 samples measured, we have: a beam NEMS made in Grenoble which was used in Ref. [33], a drum-head NEMS made in Aalto and used in Ref. [35] that we shall call "weakly-coupled", and another drum-head from Aalto (the one of this experiment), that we shall call "strongly-coupled". The beam device displayed in the first flexure large amplitude fluctuations (nicknamed "spikes") at low temperatures, described in Ref. [33]. They come from a stochastic driving force of unknown origin, that has been observed in different laboratories over the world. The first drum ("weakly-coupled") showed below about 30 mK an apparent thermal decoupling of the fundamental mode that seemed to share some of the "spikes" characteristics [61]. It was therefore impossible to measure in thermal equilibrium the mechanical modes of these structures down to (and below) 10 mK. For both devices, the microwave power required for the measurements is quite large, $n_{\rm cav}$ in the range $10^4 - 10^6$ photons confined in the cavity (see Supplementary Note 2 below for the definition of $n_{\rm cav}$).

However, the second drum could be measured down to very low powers, typically $300 - 600$ photons (using the so-called blue and red-detuned schemes, see below), thanks to its "strongly-coupled" nature. In these conditions, none of the above problems was seen (consistently with results from other labs, e.g. Ref. [57]); the remaining issues are fluctuations in the device properties themselves, which are always seen in nanomechanical experiments (as far as they are looked for), as discussed in the core of the paper (and see below, Supplementary Note 3). The link between fluctuations, "spikes", and microwave power remains mysterious, and one can only speculate on that (see Ref. [33] and Supplementary Note 3 below).

Supplementary Note 2: Single-tone microwaves

D. Chip properties

The device studied in this experiment is an aluminium drum-head (see Fig. [1] picture) of diameter 15 μm, 100 nm thickness suspended about 50 nm above a gate electrode coupled to a $\omega_c = 2\pi \times 5.7$ GHz meander microwave cavity. It is made on sapphire. The total damping of the cavity resonance is $\kappa_{\rm tot} = 2\pi \times 500$ kHz, for an external coupling of $\kappa_{\rm ext} = 2\pi \times 240$ kHz. The measure-

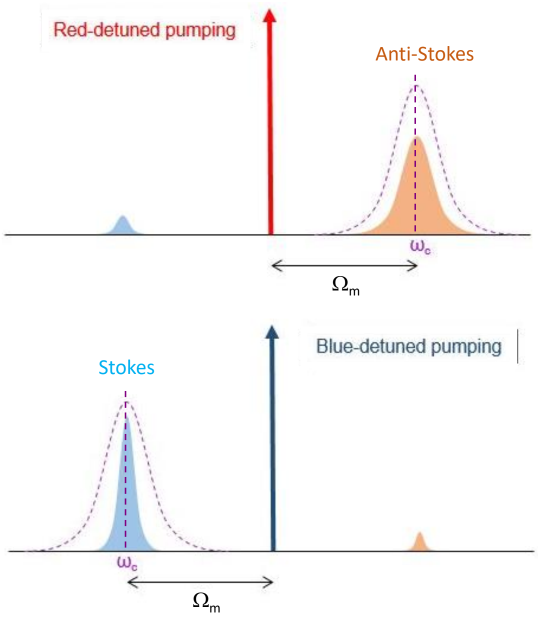

Supplementary Figure 4: **Optomechanics schemes.** Output spectra, with the cavity response depicted as dashed (not to scale). The pump tone is the vertical arrow (Dirac peak). Top: red-detuned pumping scheme. Bottom: blue-detuned pumping scheme. Only the enhanced sideband at ω_c is measured.

ment is performed in reflection, with a single coupling port; we are in the resolved sideband regime $\omega_m \gg \kappa_{tot}$.

The two lowest modes of the mechanical structure have been measured; the strongly coupled $[0,0]$ fundamental mode at $\omega_m = 2\pi \times 15.1$ MHz (for a low temperature damping of $\Gamma_m = 2\pi \times 420$ Hz), and the first non-axisymmetric mode $[0,1]$ at 25.9 MHz (for a damping of about 100 Hz) with a very small optomechanical coupling (non-zero because of the slight imperfect mode shape due certainly to the exact clamp structure). The next axisymmetric mode $[1,0]$ should be around 36 MHz but has not been characterized. From simulations, the device stores about 240 MPa stress. Mechanical damping is dominated by (rather large) clamping losses below 100 mK, which is certainly linked to the large amount of stress present in the device [66]. In our experiment, the relaxation time of the mode is thus $\tau_m = 2/\Gamma_m \approx 1$ ms, independent of temperature below 100 mK.

E. Basic calibrations

Only strict single-tone optomechanics has been used, such that the basic theory does strictly apply. This means that blue (microwave drive frequency $\omega_c + \omega_m$) and red (microwave drive frequency $\omega_c - \omega_m$) detuned pumping experiments have been performed in distinct runs. Only these two schemes have been used; see Suppl. Fig. 4 for a schematic of the techniques. The "green" pumping (pump tone applied at the cavity frequency ω_c) could not be used; below typically 50 mK, the areas of the two measured sidebands were not consistent anymore with the cryostat temperature, a feature that is observed with the "spike" problem [33]. We suspect that this is again due to the large amount of microwave power applied (which for this scheme is again on the order of 10^5 photons). This feature, which seems to affect all microwave optomechanical devices, deserves to be studied but is outside of the scope of this work.

We first characterized the optomechanical coupling g_0 by measuring at fixed temperature $T_{cryo} = 100$ mK the Brownian sideband peak as a function of applied microwave power. The peak areas and the peak widths are shown in Suppl. Figs. 5 and 6 respectively.

The lineshapes are Lorentzians (see fits Fig. [2] a insets). Basic formulas allow to link the measured peak area A (in photons/s) and linewidth ΔW (in rad/s) to the effective NEMS mode population detected n_{eff} and applied microwave power P_{in} [33, 34, 61]:

$$A = \Gamma_{opt}\, n_{eff} \quad \text{for red,} \tag{1}$$
$$A = \Gamma_{opt}\,(n_{eff}+1) \quad \text{for blue,} \tag{2}$$
$$\Delta W = \Gamma_m \pm \Gamma_{opt}, \tag{3}$$
$$\Gamma_{opt} = 4\frac{g^2}{\kappa_{tot}}, \tag{4}$$
$$g^2 = g_0^2\, n_{cav}, \tag{5}$$
$$n_{cav} = \frac{P_{in}\,\kappa_{ext}}{\hbar\omega_c\,\omega_m^2}, \tag{6}$$
$$n_{eff} = \frac{n(T)\,\Gamma_m + N_{noise}\Gamma_{opt}}{\Gamma_m \pm \Gamma_{opt}}, \tag{7}$$
$$n(T) = \frac{1}{Exp[(\hbar\omega_m)/(k_B T)] - 1}, \tag{8}$$

with the sign referring to red (+) or blue (-) detuning schemes, using the resolved-sideband condition $\kappa_{tot} \ll \omega_m$. $n(T)$ is the Bose-Einstein thermal distribution, where $T = T_{mode}$ by definition. n_{cav} is the number of drive photons confined in the cavity for the measurement. From fits (black lines) we extract the single phonon coupling $g_0 = 2\pi \times 230$ Hz.

Let us discuss these simple expressions and what they actually mean. At high temperature when $n_{eff} \gg 1$, Eqs. (1,2) tell us that the areas obtained with blue and red pumping schemes are essentially equivalent; they are just proportional to the mode population n_{eff}. However, when n_{eff} becomes very small, they start to be different: this effect known as sideband asymmetry [26], is in itself a proof that the system is very cold. In Eq. (7) which defines the effective mode population, we see that an extra term appears in addition to the Bose population: N_{noise}. This term is linked to the photon noise N_{cav} present in the microwave cavity (not to be confused with n_{cav}) through:

$$N_{noise} = N_{cav} \quad \text{for red,} \tag{9}$$
$$N_{noise} = N_{cav} + 1 \quad \text{for blue.} \tag{10}$$

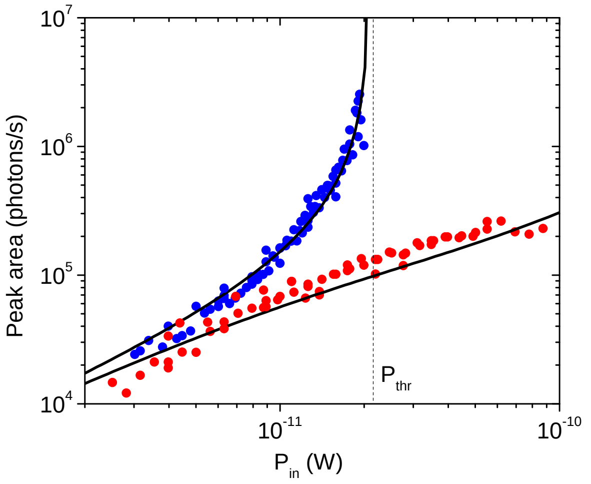

Supplementary Figure 5: **Area of sideband versus power.** Blue and red detuned measurements performed at 100 mK. Lines are fits (a slight out-of-equilibrium contribution is visible at large powers for red detuned pumping, which is discussed later in the text); the dashed vertical marks the threshold towards self-sustained oscillations (see text).

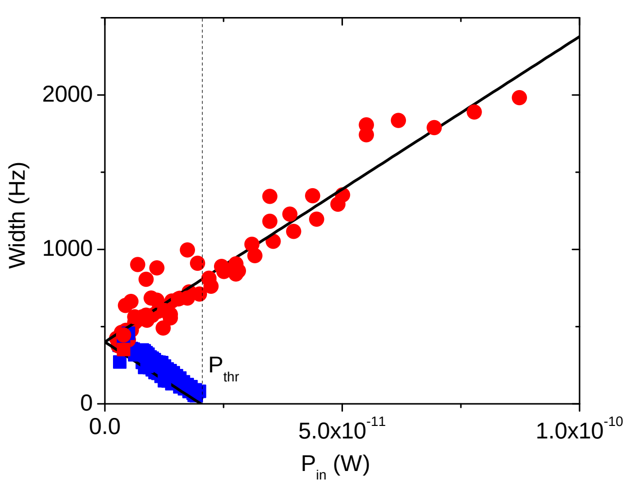

Supplementary Figure 6: **Width of sideband versus power.** Blue and red detuned measurements performed at 100 mK. Lines are fits (pointing at $\Gamma_{\rm m}$ for zero power injected); the dashed vertical marks the threshold towards self-sustained oscillations (see text).

For simplicity in our discussion, we assumed that the cavity photon noise and the one arising from the microwave port are the same. In Eq. (9), we neglected a term $(\kappa_{\rm tot}/4\omega_{\rm m})^2$ which is small in the limit of sideband resolved setups; this is actually the lower bound for red detuned active cooling fixed by quantum mechanics [34]. A generic discussion can be found in Ref. [61]. The "+1" appearing in Eq. (10) is a signature of zero point fluctuations in the cavity field [34]. If the noise $N_{\rm cav}$ was limited only by the thermal noise of the cavity, and if we take the expected electronic temperature of 40 mK to evaluate it (see Supplementary Note 4), we would obtain 10^{-3}. This is clearly negligible, but unfortunately not the whole story, as shall be discussed in Section F.

The red detuned scheme de-amplifies/cools the mode (population decreased, linewidth increased) while the blue scheme amplifies/heats (population increased, linewidth decreased). When the linewidth becomes zero (at $P_{\rm thr}$, Suppl. Figs. 5 and 6), the mechanical mode enters self-sustained oscillations [35]. This is shown in Suppl. Fig. 7. The amplitude of motion becomes then very large, and the mechanical frequency shifts because of the Duffing nonlinear effect (stretching of the membrane, Duffing coefficient about 20 Hz/nm^2, see inset) [67]. From simulations [35], we infer that the motion is in the nanometer range.

When the pump tone is not perfectly tuned, the position of the measured sideband moves linearly with applied power; an effect known as optical spring [34]. This is shown in Suppl. Fig. 8 for our experimental settings, and explains the slight difference in the actual peak positions in Fig. [2] a (but has a marginal impact on other

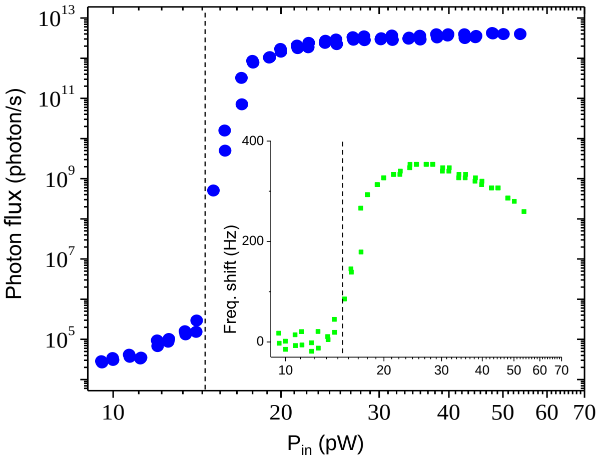

Supplementary Figure 7: **Self-sustained oscillations.** Amplitude of sideband signal as a function of power at 18 mK, in the blue detuned scheme. The dashed line marks the threshold were the linewidth becomes zero (see Suppl. Figs. 5 and 6). Inset: measured mechanical frequency shift versus power.

measured parameters). The pump position $\omega_{\rm c} \pm \omega_0$ has been kept constant over the whole experiment (with ω_0 the high temperature value of $\omega_{\rm m}$). As such, the optical spring measured was temperature-independent; the T-dependent mechanical frequency shift (Fig. [1]) being far too small compared to $\kappa_{\rm tot}$ to have any effect on this setting. But precisely because the mechanical frequency shifts with temperature, any microwave-induced physical

heating of the device would add-up and create an additional (and temperature-dependent) contribution to the effect presented in Suppl. Fig. 8 (see e.g. Ref. [33]). This is not observed in our case, and we therefore conclude that no relevant microwave-induced physical heating of the device is present, at any temperature, in the range that we explored.

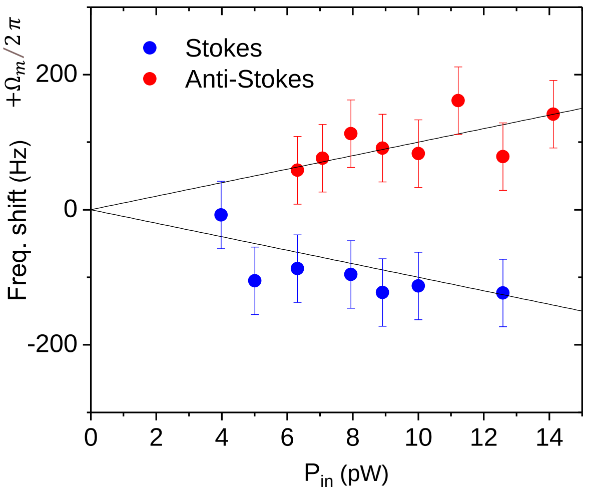

Supplementary Figure 8: **Optical spring.** Mechanical frequency shift as a function of microwave power at 15 mK for both red and blue detuned schemes. Lines are fits (see text). Error bars correspond to type A uncertainties extracted from the fitting process (Supplementary Note 3).

The point of the experiment is to use optomechanics as a probe, as little invasive as possible: ideally for extremely small injected power $n_{\rm cav} \rightarrow 0$, the amplification/de-amplification is negligible. Ideally as well, the stochastic back-action arising from the (very small, but) finite cavity/microwave port population $\Gamma_{\rm opt}\, N_{\rm noise}$ should also be essentially zero. In this limit, one would just measure a sideband signal proportional to injected power, subject only to sideband asymmetry, i.e. obtaining an area from Eqs. (1-2) proportional to $n(T)$ in the red detuned scheme, and $n(T)+1$ for blue. This leads us to define the "effective temperatures" $T_{\rm blue}, T_{\rm red}$ from the area A quoted in the core of the paper:

$$T_{\rm blue} \;=\; (n+1)\frac{\hbar\omega_{\rm m}}{k_{\rm B}} \;\; \text{blue-detuned}\;, \qquad (11)$$

$$T_{\rm red} \;=\; (n)\frac{\hbar\omega_{\rm m}}{k_{\rm B}} \qquad \text{red-detuned}. \qquad (12)$$

These are practical parameters for the experimentalist: above typically 10 mK, they both equalise to the mode temperature $T_{\rm mode}$. Having them follow $T_{\rm cryo}$ is thus a proof of thermalisation.

We therefore keep the drive power in the range 300 to 600 photons for $n_{\rm cav}$, the lowest we can afford for practical reasons. Even if small there is still a measurable amplification/de-amplification in this range; but this is

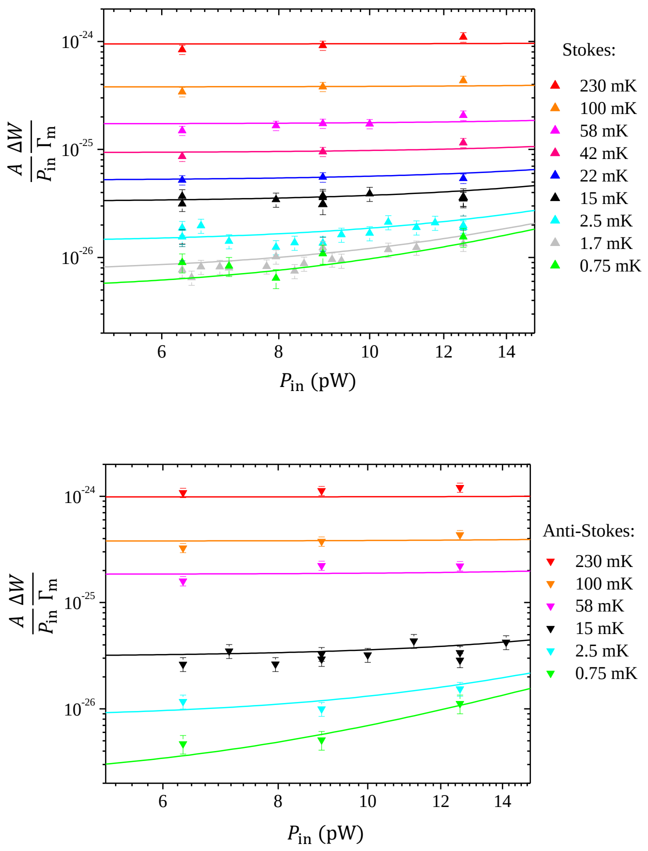

Supplementary Figure 9: **Normalised areas.** Areas normalized to injected power and linewidth as a function of $P_{\rm in}$. Top: blue-detuned pumping. Bottom: red-detuned pumping. Lines are fits explained in the text. Error bars correspond to type A uncertainties extracted from the fitting process (Supplementary Note 3).

easily corrected for since the ratio $\Gamma_{\rm opt}/\Gamma_{\rm m}$ is known (see however the discussion in Supplementary Note 3 about damping fluctuations). This is what is applied to create the main graph of Fig. [2] a. However, the sideband peaks displayed in insets have not been corrected; this is why an asymmetry is visible at high temperature between the Stokes and anti-Stokes peaks. As such, the ratio of their areas is bound at high temperature by $(\Gamma_{\rm m}-\Gamma_{\rm opt})/(\Gamma_{\rm m}+\Gamma_{\rm opt}) \approx 0.5$, as can be seen in the top inset, Fig. [2] a (sideband asymmetry thermometry). The line in the same inset graph is theory; in principle an independent proof of thermalisation down to the lowest temperatures.

F. Technical heating

Unfortunately, the second assumption discussed in the previous paragraph (ideally cold microwave mode, $N_{\rm cav}=0$) is also not fully verified: as we increase the in-

jected microwave power $P_{\rm in}$, there is technical heating of the microwave mode (to be distinguished from physical heating of the whole structure; see Suppl. Fig. 5 the red detuned data). This effect is known in the community, and not always easy to circumvent. It is presumably due to out-of-equilibrium photons that populate the cavity and act (through back-action) onto the mechanical degree of freedom. These out-of-equilibrium photons are believed to originate in the phase noise of the microwave source; an ultimate limitation of any setup. The foot of the Dirac peak of the pump "leaks" into the cavity, so to speak. But the actual mechanism behind this is not fully understood, and the magnitude of this effect does depend on sample and cool down: it has to be carefully characterized in each run.

To demonstrate this for our experiment, we plot in Suppl. Fig. 9 the area A normalised to power $P_{\rm in}$ and linewidth ΔW as a function of $P_{\rm in}$, for different temperatures $T_{\rm cryo}$. And indeed, we see at the largest power a deviation, characteristic of this effect (without which it should remain flat, and $\propto T_{\rm blue}, T_{\rm red}$). It can be fit by an out-of-equilibrium contribution $\propto (P_{\rm in})^a$ (a power law dependence with exponent a) [33], see lines in Suppl. Fig. 9. This term is independent of the scheme, and of temperature. It is of the order of 1 out-of-equilibrium photon at the lowest drive used (300 photons), and can be easily corrected for; it is negligible in all conditions presented in the core of the paper, except for two measurements performed around 700 μK (the time-trace taken with the blue scheme, Fig. [2] b, 600 photons drive, and the anti-Stokes peak Fig. [2] a, 300 photons), and the time-trace taken at 1.4 mK (blue scheme, Fig. [2] b, 600 photons drive). In these extreme cases, the correction nonetheless remains smaller than a factor two. In order to certify that there is no flaw in the analysis, a time trace with 300 photons has also been taken and processed at 14 mK; and indeed results are identical for 300 and 600 drive photons (see Supplementary Note 3 below).

As a concluding remark on the mode populations, we can compute the $n(T)$ of the nearest modes at the lowest temperature of 500 μK assuming perfect thermalization, which as discussed in the core of the paper is essentially guaranteed by our results obtained on the lowest mode (see also the discussion on thermal properties in Supplementary Note 4 below).

At this temperature the fundamental mode $[0,0]$ stores 0.3 quanta on average. The nearest non-axisymmetric mode $[0,1]$ stores about 0.1 quanta while the next axisymmetric mode $[1,0]$ has 0.03 quanta. All higher modes verify $n(T) \leq 1$ %.

Supplementary Note 3: Statistical analysis

Beyond thermalisation, a striking result of the experiment concerns the very slow, and rather large fluctuations that are seen in mechanical properties as a function of time. Obviously our measurement is not single-shot (detection background about 100 photons at 5 GHz), and it is not a QND measurement of the population ($\hat{n}$ operator); what we measure is the spectrum of the $\hat{x}$ operator, the position. We need between 5 and 20 minutes at our highest drive level in blue-detuned pumping (600 photons) in order to have an acquisition data averaged enough to be fitable (depending on temperature). This means that what we measure is a "smoothed" estimator of the time-dependent mechanical properties, but not the real-time ones. But it is an invaluable, and unique, information about whatever processes are at stake in the thermodynamical equilibrium of the device.

The setup and measurement scheme has been kept basic, so that it can be easily modelled by "conventional single-tone optomechanics". This makes the interpretation of measured data rather simple, and the only experimental parameter linked to the detection that we will have to consider for potential biases in the analysis is the (even if small) power $P_{\rm in}$ (or number of photons $n_{\rm cav}$). This has been already discussed in the previous Section. And of course, because of the nature of the data presented we have to consider carefully the potential flaws that the statistical analysis may imply. These aspects are the subject of this Appendix.

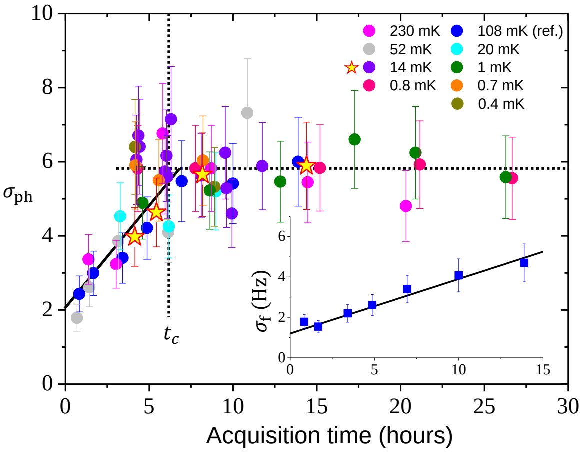

Supplementary Figure 10: **Standard deviation versus acquisition time.** Phonon standard deviation measured at different $T_{\rm cryo}$ normalised to 108 mK data, with an averaging window of 20 minutes (from 600 photon drive in blue-detuned scheme). Inset: frequency standard deviation at 108 mK. The stars are measured with 300 photons (see text). Error bars correspond to type A uncertainties extracted from the fitting process (Supplementary Note 3).

G. Potential statistical flaws

The first statistical parameter we can consider is the full acquisition time. In Suppl. Fig. 10 (main graph) we show the phonon standard deviation $\sigma_{\rm ph}$ measured at

different $T_{\rm cryo}$ scaled on the 108 mK values, as a function of the length of the acquisition time. In inset, we give the frequency standard deviation $\sigma_{\rm f}$ measured at 108 mK (damping noise is discussed in the next Section). For these data, all fits have been performed on time-traces where single points are obtained by 20 minutes averages. As explained in the core of the paper, this averaging window is sliding through the acquired set of data (which has a time step of 1 second). Apart from the highest temperatures, no peak can be seen on single files. $\sigma_{\rm ph}$ and $\sigma_{\rm f}$ have rather different behaviours, which are characteristic of the nature of their fluctuation spectra. For full acquisition times smaller than $t_{\rm c} = 5$ hours, $\sigma_{\rm ph}$ grows essentially linearly, similarly to the whole behaviour of $\sigma_{\rm f}$. This comes from the $1/f^2$ nature of the spectra. However, above 5 hours it becomes flat: this is a signature of the low-frequency cutoff $1/t_{\rm c}$ seen in the fluctuation spectrum. The standard deviation of the phonon noise is bound, and measuring with an acquisition time of 10 hours guarantees to have a proper estimate of $\sigma_{\rm ph}$. On the other hand, $\sigma_{\rm f}$ grows continuously: the spectrum is not bound at low frequency, this noise is actually non-stationary. We therefore have to quote for what acquisition length we compute $\sigma_{\rm f}$; this is what people refer to in the literature with the Allan deviation [68]. Here again, we shall compute the standard deviation with a time slot of 10 hours.

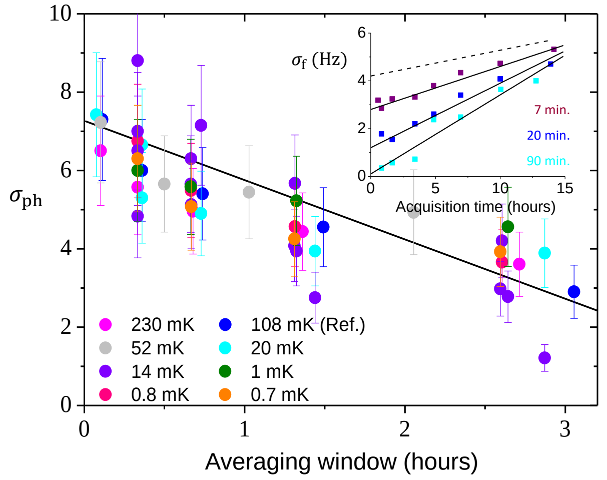

Supplementary Figure 11: **Standard deviation versus averaging window.** Phonon standard deviation at different $T_{\rm cryo}$ for acquisition times longer than 10 hours scaled on 108 mK data (line is linear guide). Inset: frequency standard deviation versus acquisition time for 3 different averaging window, at 108 mK. Lines are fits, and the dashed one is the extrapolation for zero averaging (see text). All data taken at 600 photons in blue-detuned scheme. Error bars correspond to type A uncertainties extracted from the fitting process (Supplementary Note 3).

More importantly, we have to consider the effect of the averaging window on the quoted standard deviations. In Suppl. Fig. 11 we show the effect of the size of the window on the $\sigma_{\rm ph}$ measured in the plateau region of Suppl. Fig. 10 (beyond 10 hours acquisition, scaled on the value at 108 mK). As expected, we see a rather smooth decrease of $\sigma_{\rm ph}$ when we increase the averaging time. At first order, this looks like a linear dependence; at most, when averaging 20 minutes we lose about 20 % of the total phonon fluctuation amplitude. This has been corrected for in Fig. [3] of the core of the paper. In inset on Suppl. Fig. 11, we demonstrate the impact of the averaging procedure on $\sigma_{\rm f}$; we plot it as a function of acquisition time for 3 different averaging windows. As expected, the more we average the smaller the $\sigma_{\rm f}$ value. In all cases, the dependence seems linear with respect to acquisition length. Increasing the averaging time seems to impact much more the short acquisitions (the 0-time intercept goes down very quickly by increasing the averaging), while it is less pronounced at large times. For 10 hours acquisitions, a 20 minutes averaging window decreases the impact of frequency fluctuations on the data by about 20 % (see dashed line in Suppl. Fig. 11). This has been corrected for in the $\sigma_{\rm f}(T)$ plots.

One important aspect of the measurement which also has to be discussed within the statistical analysis is the finite power at which (in blue-detuned pumping) the measurement is performed. For technical reasons, this amplifies the fluctuations: even when the average population is below one, the acquisition is performed around about 3 phonons. It is thus expected than, within our resolution all distributions shown in Fig. [2] b look Gaussian (in accord with the central limit theorem). Knowing the gain $\Gamma_{\rm m}/(\Gamma_{\rm m}-\Gamma_{\rm opt})$, one can easily recalculate the original average value. However, it is perfectly reasonable to wonder if the procedure could modify the amplitude of fluctuations (like e.g. squeeze them). We therefore performed a time-trace measurement at half the drive (300 photons), at a temperature high enough such that the averaging settings we use would still be enough to process the data over decent timescales (we took 14 mK; see stars in Suppl. Fig. 10). This leads to the orange dot in the graphs (Fig. [3] of the paper, Suppl. Fig. 12 below and stars in Suppl. Fig. 10). As one can clearly see, the obtained standard deviations are equivalent to the ones extracted with 600 photons. The overall shape of the spectra reported are also independent of microwave power and statistical analysis: the $1/f^2$ nature is absolutely robust (for both phonons and frequency/damping noises), as well as the $t_{\rm c}$ cutoff time seen for phonon fluctuations. We thus conclude that there is no flaw in the magnitude of the fluctuations reported. Also: if there was an influence from other sources of noise (especially electric noise for what concerns the optical field, damping noise impacting the area measurement or fluctuations in cryostat temperature), we would expect an effective saturation at the lowest temperatures. This is not observed, and $\sigma_{\rm ph}$ seems to follow n monotonically in Fig. [3].

In order to conclude our discussion of the statistical analysis, we shall comment on the relevant timescales

involved in the experiment. The physical filtering is about 1 Hz from the lock-in, and the averaging extrapolation is performed from a typical time of a minute. Therefore, our analysis is a proper treatment of the data that reproduces genuinely only the slow component of the population fluctuations. To be concrete, the inset of Fig. [1] suggests that the mechanical damping writes $\Gamma_m = \Gamma_{ph} + \Gamma_{TLS}$, with $\Gamma_{ph} \gg \Gamma_{TLS}$ (and thus $\Gamma_m \approx \Gamma_{ph}$). The phonon contribution is by far too fast compared to our measurement; its contribution to the statistics is suppressed by a factor of the order of 10 s/1 ms. On the other hand, the TLS contribution Γ_{TLS} (presumably of order $1/t_c$) is slow enough to be perfectly reproduced by our analysis: this is the genuine mechanism that has to underlie our results. Also by construction, our data actually contain all possible sources of slow noise integrated together (in particular the impact of $1/f$-like electronic noise, the $1/f^2$ damping noise discussed below, and even the fridge long-term temperature instability). It is also true for the frequency fluctuations. One could therefore guess that our σ_{ph}, σ_f could only possibly overestimate genuine TLS- fluctuations, if any imperfection is to be claimed. For this reason, it is rather surprising to measure a sub-Poissonian statistics; it would have been naively more natural to expect a result above the $\sigma_{ph} = \sqrt{n}$ dependence. We thus conclude that the prefactor 0.5 in the $\sigma_{ph} = 0.5\sqrt{n}$ law provides important insight into the TLS bath and the way in which it interacts with the mechanics.

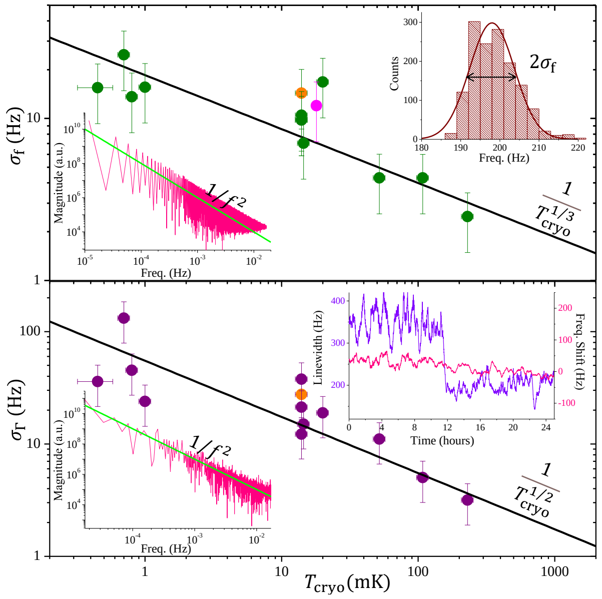

Supplementary Figure 12: **Damping noise.** Comparing frequency (top) and damping (bottom) fluctuations as a function of temperature (standard deviation σ_Γ computed for 10 h acquisition, as for σ_f). The magenta point is inferred from the self-oscillating state measurements (see Suppl. Fig. 7 above), in agreement with the Brownian motion result (also on Fig. [3] of the paper at 18 mK). Insets: Gaussian like statistics and $1/f^2$ spectra. In the last inset, we report on a damping sudden jump, while nothing is seen on the frequency. The black lines in the main graphs are power laws $1/(T_{cryo})^a$ guides to the eye. The orange dots, as in other graphs, are obtained at half the drive (see text). Error bars correspond to type A uncertainties extracted from the fitting process (Supplementary Note 3).

H. Frequency and damping fluctuations

Besides frequency and area fluctuations, we also report on damping fluctuations. These are usually not looked at, but are always present in nanomechanical systems [52]. Actually, all the essential characteristics of the frequency noise ($1/f^2$ spectrum, Gaussian-like distribution, rare telegraph-like jumps, and $1/T^a$ temperature dependence below 200 mK) appear to be shared by damping noise. However, these two noises seem to be not (at least perfectly) correlated. This is demonstrated in Suppl. Fig. 12.

Even if not strictly equivalent, the frequency noise looks quite similar to what is found in superconductors: $1/f^a$-like, growing with decreasing temperature [50]. Also, the damping noise seems to dominate over other sources of errors (namely the amplifier noise, and then the fit error) when evaluating mechanical parameters, and is thus responsible for the scatter in the data. We can only speculate on the origin of all material-dependent fluctuations, namely "spikes" [33], frequency noise, and damping noise. These may be related to the same underlying mechanisms, but it will require further work to be proven. Two-Level-Systems (TLS) are often invoked when dealing with these issues, since they explain the frequency shifts $\omega_m(t) - \omega_0$ as well as the high temperature dependence of the damping Γ_m. The exact fit function (dashed line in Fig. [1]) for the frequency shift reads $\omega_m(T) - \omega_0 \propto (Re[\Psi(1/2 + (\hbar\omega_0)/(2\pi i k_B T))] - Ln[(\hbar\omega_0)/(k_B T)])$ with Ψ the Digamma function [42]. This leads to the observed logarithmic-like dependence (full line in Fig. [1]). The damping happens to be linear with temperature at high temperature, a feature also observed for other NEMS devices in the same temperature range [69]. An intriguing effect has been noticed in the mechanical frequency shift at the lowest temperatures (below 10 mK): even though the equilibrium temperature follows the Log (or equivalently Digamma) law, we discovered temperature hysteresis when ramping up the temperature after cooling. This could be the signature of some sort of "glassy" behaviour within the TLS bath (subject to TLS-TLS interactions? See e.g. Ref. [50, 70]), but we are again limited to speculations and one would need further investigations to understand this. More discussions can be found in Ref. [61].

Supplementary Note 4: Thermal modeling

The experimental work presented in the paper is about the thermalisation of a mesoscopic mechanical object down to 500 μK. We present in this Appendix the basic thermal modeling of the device, that consistently supports our experimental result. First, we will review the fundamental models to calculate the specific heat (Debye model) and the thermal transport (Kinetic equation and Casimir model). Basic material-dependent parameters will be reminded.

Nomenclature:

- k thermal conductivity W.m^{-1}.K^{-1}
- K thermal conductance W.K^{-1}
- $c_{\rm p}$ specific heat in J.m^{-3}.K^{-1}
- C heat capacity J.K^{-1}
- Λ phonon mean-free-path

The modeling is kept basic, since only orders of magnitudes are sought. As such, we should point out already that the specific heat of materials is not affected by small dimensions at the 100-nanometer scale (we can thus keep using bulk values) [71]. On the other hand, for the thermal conductance we need to take into account the small dimensions of the heat conductor (and thus renormalize the bulk values and adapt them to our 2D-membrane sample).

I. Thermal properties of superconducting aluminium membrane

The heat carriers that will matter for calculating the characteristic thermalization time (and other parameters) are the phonons. Indeed, thermal transport in Aluminium at low enough temperature is dominated by phonons and not any more by electrons. The experiment is performed below 200 mK: the temperature is then far lower than the superconducting critical temperature $T_{\rm c}$ of aluminium ($T_{\rm c} \approx 1.2$ K); we can thus consider that all the conducting electrons are BCS-condensed, and consequently the thermal transport will happen through the lattice only (phonons), because the condensed electrons cannot carry entropy (then do not contribute to thermal transport) [72]. This can be seen in the thermal transport measurements done by Zavaritskii [73] presented in Suppl. Fig. 13 where the thermal conductivity dramatically changes between $T_{\rm c}$ and $T_{\rm c}/5$: below 0.2 K one sees that only the phonon contribution remains as it varies like T^3.

As well, the contribution of electrons to the specific heat $c_{\rm p}$ is decreasing exponentially below $T_{\rm c}$; this is a standard result that can also be calculated in BCS-theory [72]. We can thus also neglect electrons in the specific heat, and consider only lattice vibrations for the heat capacity of the object. We shall come back at the end of this Appendix to electronic properties.

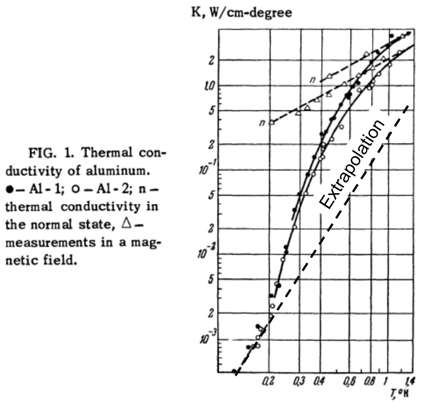

Supplementary Figure 13: **Thermal conductance.** Measurement done by Zavaritskii [73] in 1958 of thermal conductivity in aluminium, on bulk materials (few centimeter large). The extrapolation of that curve and the corresponding fit are given in Suppl. Fig. 15 (and Suppl. Tab. 1). Image adapted from Ref. [73].

Material	$v_{\rm s}$	$\theta_{\rm D}$	ρ	$c_{\rm p}$	$k_{\rm bulk}$
Al	6700	468	2 700	0.41 $\times T^3$	23.4 $\times T^3$

Supplementary Table 1: Aluminium (Al) data for thermal properties: the speed of sound $v_{\rm s}$ in m/s, the mass density ρ in kg/m^3, the Debye temperature $\theta_{\rm D}$ (in K). Specific heat in J.m^{-3}.K^{-1} and (bulk) thermal conductivity in W.m^{-1}.K^{-1}.

J. Specific heat of the Al membrane

We will use the Debye model to calculate the phonon specific heat of aluminium associated to the crystal lattice, in other terms the phonon specific heat. For $T \ll \theta_{\rm D}$ (the Debye temperature), $c_{\rm p}$ is given by the following equation using the averaged speed of sound $v_{\rm s}$:

$$c_{\rm p} = \frac{2\pi^2}{5} \frac{k_{\rm B}^4}{\hbar^3 v_{\rm s}^3} T^3. \qquad (13)$$

The specific heat is in J.m^{-3}.K^{-1}. To convert it to J.kg^{-1}.K^{-1}, if necessary, one has to divide Eq. (13) by the density ρ in kg.m^{-3} (see e.g. Ref. [71]). Parameters are given in Suppl. Tab. 1 with the thermal conductance fit on Ref. [73] (see Suppl. Fig. 15). We find a specific heat for the aluminium crystal lattice of $c_{\rm p} = 0.41 \times T^3$ J.m^{-3}.K^{-1}.

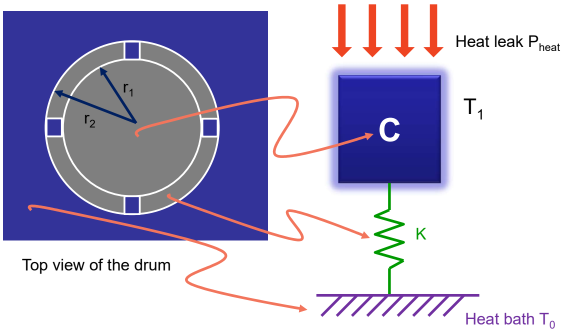

Supplementary Figure 14: **Thermal model.** Left: schematics (not to scale) of the suspended aluminium disk (at temperature T_1) and the (cut) torus connecting the disk to the heat bath (the sapphire substrate, at T_0), see image in Fig. [1]. Right: thermal model of the suspended disk membrane of heat capacity $C \approx C_{\rm membrane}$ and thermal conductance $K \approx K_{\rm torus}$ of the torus (we neglect then the small cuts). "heat leak" stands for all the heat sources acting on the membrane (see discussion in text).

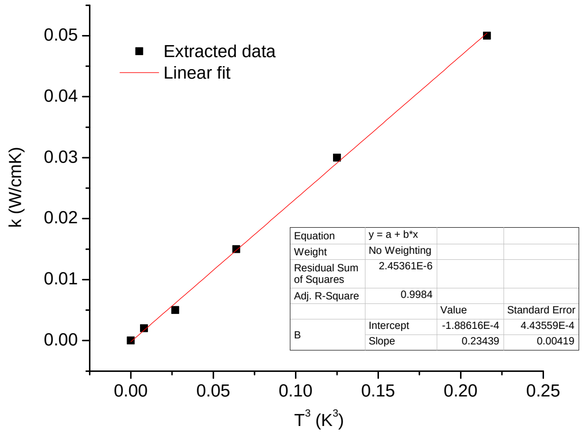

Supplementary Figure 15: **Thermal conductivity fit.** Bulk thermal conductivity of aluminium as a function of T^3 extrapolated from Zavaritskii [73] up to high temperatures enabling to calculate the power law $k \approx 23.4\, T^3$ W.m^{-1}.K^{-1} (Suppl. Tab. 1).

K. Thermal conductivity, Kinetic equation and phonon mean-free-path in the structure

The second important calculation is about estimating the thermal conductance of the structure in order to evaluate its thermalisation time (and potential thermal gradients). The simplified model we consider is shown in Suppl. Fig. 14.

The phonon thermal conductance is related to specific heat and phonon mean-free-path through the kinetic equation (coming from the Boltzmann transport equation at low temperature, see e.g. Ref. [71]):

$$k = \frac{1}{3} c_{\rm p} \Lambda v_{\rm s}. \tag{14}$$

From this equation, we can calculate the bulk phonon mean-free-path Λ if we know the bulk (experimental) thermal conductivity k and the specific heat $c_{\rm p}$. The measured bulk thermal conductivity is taken from Zavaritskii [73] (Suppl. Fig. 13) by fitting the lower part of the curve below 0.2 K (see Suppl. Tab. 1; and the power law extrapolation in Suppl. Fig. 15). This gives a mean-free-path for bulk phonons of $\Lambda = 2.5 \times 10^{-2}$ m (2.5 cm, and independent of temperature since both k and $c_{\rm p}$ have the same T^3 dependence). From this result, we shall simplify the problem by considering the membrane as isothermal at temperature T_1, and assume that the temperature gradient (if any) happens at the torus region. We should now define the thermal conductance of this zone.

When we build a system where one dimension is smaller than this mean-free-path, the phonons will scatter on the rough surfaces that confine them; then Λ will be reduced. The new mean-free-path can be obtained following the Casimir model [74, 76, 77], a rather simple model, where we assume that the phonons are fully scattered on surfaces (a sort of "phonon black body"). This is indeed what happens in the torus region, where the thickness $e_{\rm p}$ is only about 100 nm. Then the new ("confined") $\Lambda_{\rm torus}$ is essentially given by the smallest dimension of the thermal conduction channel (a "worse estimate", here the thickness already quoted, much smaller than the radii r_1 and r_2). Taking as an order-of-magnitude approximation $\Lambda_{\rm torus} \approx e_{\rm p}$ (then 10^4 times smaller than the bulk one), the "confined" thermal conductivity writes:

$$k_{\rm Al\, nano} = \frac{1}{3} c_{\rm p} \Lambda_{\rm torus} v_{\rm s}, \tag{15}$$

where $c_{\rm p}$ is the bulk specific heat in J.m^{-3}.K^{-1}, and $\Lambda_{\rm torus}$ the new phonon mean-free-path in the Al torus. This is giving a thermal conductivity of $k_{\rm Al\, nano} = 10^{-4}\, T^3$ W.m^{-1}.K^{-1}.

L. Characteristic thermalisation time

The characteristic thermalisation time $\tau_{\rm th}$ is given by $\tau_{\rm th} = C_{\rm membrane}/K_{\rm torus}$. We define the membrane heat capacity:

$$C_{\rm membrane} = c_{\rm p} V, \tag{16}$$

with $V = e_{\rm p} \pi r_1^2$ the volume of the suspended part in Suppl. Fig. 14 (with $r_1 = 7$ μm). The numerical value is then $C_{\rm membrane} = 6 \times 10^{-18}\, T^3$ J.K^{-1}. In order to estimate the thermal conductance $K_{\rm torus}$ of the Al membrane to the heat bath, we make a simple "worse estimate" calculation assuming that the thermal gradient develops on

the full width of the torus, from r_1 to r_2 (see Suppl. Fig. 14). Since $r_1, r_2 \gg \Lambda_{\rm torus}$, we shall assume that the conduction is diffusive in the torus. Noting that the dominant phonon wavelength $\lambda_{\rm dom.} \approx 2.23\,\hbar v_{\rm s}/(k_{\rm B}T)$ is of the order of $e_{\rm p}$ around 1 K, we can treat the problem as pure 2D transport, leading to the simple result [78]:

$$K_{\rm torus} = \frac{2\pi e_{\rm p}}{ln(r_2/r_1)} k_{\rm Al\,nano}, \tag{17}$$

with the two radii r_1 =7 μm and r_2 =10 μm. We thus get as an estimate $K_{\rm torus} = 1.6 \times 10^{-10}\,T^3$ W.K^{-1}.

We obtain from our estimates $\tau_{\rm th} = 4 \times 10^{-8}$ s (that is 40 nanoseconds), independent of temperature. This is obviously a rough estimate, giving however the correct order of magnitude. It tells us that the bath itself can accept/give thermal energy at a very fast rate, up to around a GHz.

M. Kapitza thermal resistance and heat dissipation within the aluminium

The simple model assumes that the sapphire substrate and the copper cell (which are macroscopic) are well anchored at the cryostat temperature T_0. But there is an extra thermal boundary resistance that we did not include yet: the Kapitza resistance between the aluminum torus and the sapphire, which is due to the acoustic mismatch between the two solids [65]. We take from Swartz *et al.* [75] the thermal conductance per surface given by: $k_{\rm ac.\,mis.} = 0.1\,T^3$ W.cm^{-2}.K^{-1}, then $K_{\rm Kapitza} = k_{\rm ac.\,mis.} \times S_{\rm torus}$ with $S_{\rm torus}$ the area of the torus. From our dimensions, we calculate a contact surface $S_{\rm torus} = 1.6 \times 10^{-6}$ cm^2, which leads to a Kapitza thermal conductance of $1.6 \times 10^{-7}\,T^3$ W.K^{-1}. This value is much larger than $K_{\rm torus}$, which means that we can indeed safely neglect it in our discussion.

At the lowest temperatures, a thermal gradient might develop between the bulk (at temperature T_0) and the aluminum drum-head (at T_1):

$$T_1 - T_0 = P_{\rm heat}/K_{\rm torus}, \tag{18}$$

with $P_{\rm heat}$ the total power that is absorbed by the suspended membrane (Suppl. Fig. 14). The question that naturally arises is: what mechanisms eventually limit $P_{\rm heat}$? Talking only about known (and somehow steady, thus not considering the "spike" issue here) mechanisms, one immediately thinks of the microwave power injected, or the imperfect r.f. filtering that reaches the sample. The former is measured to be negligible (see Supplementary Note 2), while the latter couples essentially to the electrons (see following subsection), not to the phonons. Black-body radiation is also negligible, since the whole chip is enclosed in a copper cell thermalised to the nuclear stage, which acts as a shield. Heating due to cosmic rays or natural radioactivity (from the building itself) is also an eligible mechanism [79], although almost impossible to estimate. Since the cross section of the device (and its thickness) is extremely small, we shall also neglect it.

Beyond radiation, one can also think about adsorption of gas particles: using ^{4}He as exchange gas in the 4 K pre-cooling process, if the remaining vacuum in the can is not low enough at cryogenic temperatures, atoms may desorb from hot parts and condense on the cold ones (i.e. the chip), creating thus an almost permanent heat leak [65]. This phenomenon is well known, and has been taken care of. Finally, the last external mechanism that people take seriously in consideration is mechanical vibrations. This is known to be detrimental to low frequency devices, generating huge heat loads if not properly handled by complex suspension systems [80]. For high frequency devices, this seems to be much less of a problem: they do thermalise to tens of milliKevin with conventional cryogenics [3]. In our case, the whole cryostat is suspended on air-mounts with about a ton of concrete anchoring, which is required for cryogenic purposes (the copper stage shall not vibrate in the 7 T field, otherwise eddy currents would create a huge heat leak) [65].

We are therefore left with internal sources of heat within the Al membrane, which can become (very) relevant at ultra-low temperatures [65]. These are time-dependent (but with rather long time-scales, so that they always contribute to some extent), and originate in H_2 inclusions, structural defects (e.g. grain boundaries in the metal layer, or TLSs in glasses) that can relax, or even radioactive impurities (citing documented effects). It is impossible to estimate quantitatively this contribution, apart from fitting results from an actual experiment. We shall thus simply consider a reasonable upper bound that supports our finding: about 0.1 nW/kg (after a few weeks of experiments below 1 K) [65]. This number corresponds to a negligible heating of the mechanical element above 1 mK, and about 100 μK temperature increase only around 600 μK.

N. Conduction electrons

We shall conclude our discussion on thermal properties by a quick look at the electron bath in the (superconducting) aluminium. The electron bath decouples from the phonon bath in the membrane following the equation [85]:

$$P_{\rm e\text{-}} = V g_{\rm e\text{-}ph} \left(T_{\rm e\text{-}}^5 - T_{\rm ph}^5\right), \tag{19}$$

where $P_{\rm e\text{-}}$ is the power arriving on the electrons, $T_{\rm e\text{-}}$ and $T_{\rm ph}$ are respectively the electronic and phononic temperatures, and V the volume of the drum. $g_{\rm e\text{-}ph}$ is the electron-phonon coupling constant, taken here to be 0.4×10^9 W.K^{-5}m^{-3} [86]. From the discussion of the previous section, we can take $T_{\rm ph} = T_1 \approx T_0$.

Since these electrons hold a vanishingly small specific heat [72], any small source of heat $P_{\rm e\text{-}}$ easily keeps them hot: this is a major problem in quantum electronics,

which required a lot of work over the years to prevent superconducting circuits to be poisoned by quasi-particles (i.e. non-paired electrons) [47]. As an illustration here, a rather correct filtering of r.f. noise leading to a heat leak of 1 fW would create a base temperature for the electrons of 40 mK, while phonons do cool below 1 mK. An extremely good filtering producing $P_{e^-} \approx 1$ aW would still keep T_{e^-} at about 10 mK, among the best electronic temperatures reported in nanostructures.

---

[1] A. J. Leggett, Testing limits of quantum mechanics: motivation, state of play, prospects, J. Phys.: Condens. Matter Vol. 14, pp. R415-R451 (2002).

[2] A. J. Leggett, Macroscopic quantum systems and the quantum theory of measurement, Suppl. of the Progr. of Theor. Phys. Vol. 69, pp. 80 (1980).

[3] J. D. Teufel, T. Donner, Dale Li, J. W. Harlow, M. S. Allman, K. Cicak, A. J. Sirois, J. D. Whittaker, K. W. Lehnert, and R. W. Simmonds, Sideband cooling of micromechanical motion to the quantum ground state, Nature Vol. 475, pp. 359-363 (2011).

[4] Jasper Chan, T. P. Mayer Alegre, Amir H. Safavi-Naeini, Jeff T. Hill, Alex Krause, Simon Gröblacher, Markus Aspelmeyer and Oskar Painter, Laser cooling of a nanomechanical oscillator into its quantum ground state, Nature Vol. 478, pp. 89-92 (2011).

[5] E. Verhagen, S. Deléglise, S. Weis, A. Schliesser and T. J. Kippenberg, Quantum-coherent coupling of a mechanical oscillator to an optical cavity mode, Nature Vol. 482, pp. 63-67 (2012).

[6] Uros Delic, Manuel Reisenbauer, Kahan Dare, David Grass, Vladan Vuletic, Nikolai Kiesel, Markus Aspelmeyer, Cooling of a levitated nanoparticle to the motional quantum ground state, Science Vol. 367, pp. 892895 (2020).

[7] C. F. Ockeloen-Korppi, E. Damskägg, J.-M. Pirkkalainen, M. Asjad, A. A. Clerk, F. Massel, M. J. Woolley and M. A. Sillanpää, Stabilized entanglement of massive mechanical oscillators, Nature Vol. 556, Issue 26, pp. 478-482 (2018).

[8] Ralf Riedinger, Andreas Wallucks, Igor Marinkovic, Clemens Löschnauer, Markus Aspelmeyer, Sungkun Hong and Simon Gröblacher, Remote quantum entanglement between two micromechanical oscillators, Nature Vol. 556, Issue 26, pp. 473477 (2018).

[9] Angelo Bassi, Kinjalk Lochan, Seema Satin, Tejinder P. Singh, Hendrik Ulbricht, Models of wave-function collapse, underlying theories, and experimental tests, Reviews of Modern Physics, Vol. 85, pp. 471-526 (2013).

[10] William Marshall, Christoph Simon, Roger Penrose, and Dik Bouwmeester, Towards Quantum Superpositions of a Mirror, Phys. Rev. Lett. Vol. 91, Issue 13, 130401 (2003).

[11] R. Penrose, On gravitys role in quantum state reduction, General Relativity and Gravitation Vol. 28, pp. 581 (1996).

[12] S. Vinjanampathy and J. Anders, Quantum thermodynamics, Contemp. Phys. Vol. 57, pp. 545579 (2016).

[13] P.C.E. Stamp, Environmental decoherence vs. intrinsic decoherence, Phil. Trans. Roy. Soc. A370, pp. 4429, (2012).

[14] E. Joos, H.D. Zeh, C. Kiefer, D. Giulini, J. Kupsch, and I.-O. Stamatescu, *Decoherence and the appearance of a classical world in quantum theory*, Springer, Second edition (2003).

[15] Alberto Ronzani, Bayan Karimi, Jorden Senior, Yu-Cheng Chang, Joonas T. Peltonen, ChiiDong Chen and Jukka P. Pekola, Tunable photonic heat transport in a quantum heat valve, Nature Physics Vol. 14, pp. 991-995 (2018).

[16] F. Brange, P. Samuelsson, B. Karimi, and J. P. Pekola, Nanoscale quantum calorimetry with electronic temperature fluctuations, Phys. Rev. B Vol. 98, 205414 (2018).

[17] A. A. Clerk, Quantum-limited position detection and amplification: A linear response perspective, Phys. Rev. B Vol. 70, 245306 (2004).

[18] M. D. LaHaye, O. Buu, B. Camarota, K. C. Schwab, Approaching the Quantum Limit of a Nanomechanical Resonator, Science Vol. 304, Issue 5667, pp. 74-77 (2004).

[19] S. Etaki, M. Poot, I. Mahboob, K. Onomitsu, H. Yamaguchi, and H. S. J. Van Der Zant, Motion detection of a micromechanical resonator embedded in a d.c. SQUID. Nature Physics Vol. 4, pp. 785 (2008).

[20] V.B. Braginsky and A.B. Manukin, *Measurement of weak forces in physics experiments*, University of Chicago press (1977).

[21] O. Arcizet, P.-F. Cohadon, T. Briant, M. Pinard and A. Heidmann, Radiation-pressure cooling and optomechanical instability of a micromirror, Nature Vol. 444, pp. 71-74 (2006).

[22] P.B. Abbott et al. & (LIGO Scientific Collaboration and Virgo Collaboration), GW150914: First results from the search for binary black hole coalescence with Advanced LIGO, Physical Review D Vol. 93, 122003 (2016).

[23] C. A. Regal, J. D. Teufel, and K. W. Lehnert, Measuring nanomechanical motion with a microwave cavity interferometer, Nature Physics Vol. 4, pp. 555 (2008).

[24] A. A. Clerk, M. H. Devoret, S. M. Girvin, Florian Marquardt, R. J. Schoelkopf, Introduction to quantum noise, measurement, and amplification, Reviews of Modern Physics, Vol. 82, pp. 1155-1208 (2010).

[25] J.-M. Pirkkalainen, S. U. Cho, Jian Li, G. S. Paraoanu, P. J. Hakonen and M. A. Sillanpää, Hybrid circuit cavity quantum electrodynamics with a micromechanical resonator, Nature Vol. 494, pp. 211-215 (2013).

[26] A. J. Weinstein, C. U. Lei, E. E. Wollman, J. Suh, A. Metelmann, A. A. Clerk, and K. C. Schwab, Observation and Interpretation of Motional Sideband Asymmetry in a Quantum Electromechanical Device, Phys. Rev. X Vol. 4, 041003 (2014).

[27] V.B. Braginsky, A.B. Manukin, M.Y. Tikhonov, Investigation of dissipative ponderomotive effects of electromagnetic radiation, J. of Experimental and Theoretical Physics Vol. 58, 1549-1552 (1970).

[28] Mingyun Yuan, Vibhor Singh, Yaroslav M. Blanter, Gary A. Steele, Large cooperativity and microkelvin cooling with a three-dimensional optomechanical cavity, Nat. Comm. Vol. 6, 8491 (2015).

[29] A.D. OConnell, M. Hofheinz, M. Ansmann, Radoslaw C.

Bialczak, M. Lenander, Erik Lucero, M. Neeley, D. Sank, H. Wang, M. Weides, J. Wenner, John M. Martinis, and A. N. Cleland, Quantum ground state and single phonon control of a mechanical resonator, Nature Vol. 464, pp. 697 (2010).

[30] Martin V. Gustafsson, Thomas Aref, Anton Frisk Kockum, Maria K. Ekström, Göran Johansson, Per Delsing, Propagating phonons coupled to an artificial atom, Science Vol. 346, Issue 6206, pp. 207-211 (2014).

[31] Gregory S. MacCabe, Hengjiang Ren, Jie Luo, Justin D. Cohen, Hengyun Zhou, Alp Sipahigil, Mohammad Mirhosseini, Oskar Painter, Nano-acoustic resonator with ultralong phonon lifetime, Science Vol. 370, Issue 6518, pp. 840-843 (2020).

[32] George Pickett and Christian Enss, The European Microkelvin Platform, Nature Reviews Materials Vol. 3, 18012 (2018).

[33] X. Zhou, D. Cattiaux, R. R. Gazizulin, A. Luck, O. Maillet, T. Crozes, J.-F. Motte, O. Bourgeois, A. Fefferman, and E. Collin, On-chip Thermometry for Microwave Optomechanics Implemented in a Nuclear Demagnetization Cryostat, Phys. Rev. Applied Vol. 12, 044066 (2019).

[34] Markus Aspelmeyer, Tobias J. Kippenberg, Florian Marquardt, Cavity optomechanics, Reviews of Modern Physics, Vol. 86, pp. 1391-1452 (2014).

[35] D. Cattiaux, X. Zhou, S. Kumar, I. Golokolenov, R. R. Gazizulin, A. Luck, L. Mercier de Lépinay, M. Sillanpää, A. D. Armour, A. Fefferman, and E. Collin, Beyond linear coupling in microwave optomechanics, Phys. Rev. Research Vol. 2, 033480 (2020).

[36] B. D. Hauer, P. H. Kim, C. Doolin, F. Souris, and J. P. Davis, Two-level system damping in a quasi-one-dimensional optomechanical resonator, Phys. Rev. B Vol. 98,214303 (2018).

[37] Garrett D. Cole, Ignacio Wilson-Rae, Katharina Werbach, Michael R. Vanner and Markus Aspelmeyer, Phonon-tunnelling dissipation in mechanical resonators, Nature Comm. Vol. 2, 231 (2011).

[38] I. Wilson-Rae, R. A. Barton, S. S. Verbridge, D. R. Southworth, B. Ilic, H. G. Craighead, and J. M. Parpia, High-Q Nanomechanics via Destructive Interference of Elastic Waves, Phys. Rev. Lett. Vol. 106, 047205 (2011).

[39] Matthias Imboden and Pritiraj Mohanty, Evidence of universality in the dynamical response of micromechanical diamond resonators at millikelvin temperatures, Phys. Rev. B Vol. 79, 125424 (2009).

[40] K. J. Lulla, M. Defoort, C. Blanc, O. Bourgeois, and E. Collin, Evidence for the Role of Normal-State Electrons in Nanoelectromechanical Damping Mechanisms at Very Low Temperatures, Pys. Rev. Lett. Vol. 110, 177206 (2013).

[41] A. D. Fefferman, R. O. Pohl, and J. M. Parpia, Elastic properties of polycrystalline Al and Ag films down to 6 mK, Phys. Rev. B Vol. 82, 064302 (2010).

[42] W. A. Phillips, Two-level states in glasses, Rep. Prog. Phys. Vol. 50, pp. 1657-1708 (1987).

[43] N. Brahms, T. Botter, S. Schreppler, D.W.C. Brooks and D.M. Stamper-Kurn, Optical Detection of the Quantization of Collective Atomic Motion, Physical Review Letters Vol. 108, 133601 (2012).

[44] R. Landauer, The noise is the signal, Nature Vol. 392, pp. 658659 (1998).

[45] L.D. Landau and E.M. Lifshitz, *Statistical Physics* 3rd Ed., Elsevier Ltd. (1980).

[46] E. Nazaretski, R. D. Merithew,V.O. Kostroun, A.T. Zehnder, R.O. Pohl, and J.M. Parpia, Effect of Low-Level Radiation on the Low Temperature Acoustic Behavior of a-SiO_2, Phys. Rev. Lett. Vol. 92, 245502 (2004).

[47] E. T. Mannila, P. Samuelsson, S. Simbierowicz, J. T. Peltonen, V. Vesterinen, L. Grönberg, J. Hassel, V. F. Maisi, and J. P. Pekola, A superconductor free of quasiparticles for seconds, arXiv:2102.00484v1 (2021).

[48] T.C.P. Chui, D.R. Swanson, M.J. Adriaans, J.A. Nissen, J.A. Lipa, Temperature Fluctuations in the Canonical Ensemble, Phys. Rev. Lett. Vol. 69, no 21, pp. 3005 (1992).

[49] H.J. Carmichael, Statistical Methods in Quantum Optics 1, Springer-Verlag Berlin Heidelberg (1999).

[50] J. Burnett, L. Faoro, I. Wisby, V.L. Gurtovoi, A.V. Chernykh, G.M. Mikhailov, V.A. Tulin, R. Shaikhaidarov, V. Antonov, P.J. Meeson, A. Ya. Tzalenchuk and T. Lindström, Evidence for interacting two-level systems from the $1/f$ noise of a superconducting resonator, Nat. Comm. Vol. 5, 4119 (2014).

[51] Clemens Müller, Jared H Cole and Jürgen Lisenfeld, Rep. Prog. Phys. Vol. 82, 124501 (2019).

[52] Olivier Maillet, Xin Zhou, Rasul R. Gazizulin, Rob Ilic, Jeevak M. Parpia, Olivier Bourgeois, Andrew D. Fefferman, and Eddy Collin, Measuring Frequency Fluctuations in Nonlinear Nanomechanical Resonators, ACS Nano Vol. 12, Issue 6, pp. 57535760 (2018).

[53] M. Bartkowiak, M. Bazrafshan, C. Fischer, A. Fleischmann, C. Enss, Nuclear Quadrupole Moments as a Microscopic Probe to Study the Motion of Atomic Tunneling Systems in Amorphous Solids, Phys. Rev. Lett. Vol. 110, 205502 (2013).

[54] Olivier Maillet, Xin Zhou, Rasul Gazizulin, Ana Maldonado Cid, Martial Defoort, Olivier Bourgeois, and Eddy Collin, Non-linear Frequency Transduction of Nanomechanical Brownian Motion, Phys. Rev. B Vol. 96, 165434 (2017).

[55] Yaxing Zhang and M. I. Dykman, Spectral effects of dispersive mode coupling in driven mesoscopic systems, Phys. Rev. B Vol. 92, 165419 (2015).

[56] Carlton M. Caves, Kip S. Thorne, Ronald W.P. Drever, Vernon D. Sandberg, and Mark Zimmermann, On the measurement of a weak classical force coupled to a quantum-mechanical oscillator. I. Issues of principle, Rev. of Mod. Phys. Vol. 52, pp. 341 (1980).

[57] J. Suh, A. J. Weinstein, C. U. Lei, E. E. Wollman, S. K. Steinke, P. Meystre, A. A. Clerk, K. C. Schwab, Mechanically Detecting and Avoiding the Quantum Fluctuations of a Microwave Field, Science Vol. 344, Issue 6189, pp. 1262-1265 (2014).

[58] J. D. Teufel, T. Donner, M. A. Castellanos-Beltran, J. W. Harlow and K. W. Lehnert, Nanomechanical motion measured with an imprecision below that at the standard quantum limit, Nature Nanotechnology Vol. 4, pages 820823 (2009).

[59] A.D. Armour and M.P. Blencowe, Probing the quantum coherence of a nanomechanical resonator using a superconducting qubit: I. Echo scheme, New J. Phys. Vol. 10, 095004 (2008).

[60] William Marshall, Christoph Simon, Roger Penrose, and Dik Bouwmeester, Towards Quantum Superpositions of a Mirror, Phys. Rev. Lett. Vol. 91, 130401 (2003).

[61] D. Cattiaux, *Ultra-low temperatures microwave optomechanics for quantum sensing*, PhD thesis (2021).

[62] C. Bäuerle, Y. Bunkov, S.N. Fisher, Chr. Gianese and H. Godfrin, The new Grenoble 100 microKelvin refrigerator, Czekoslovak J. of Phys. **46**, suppl S5, 2791-2792, (1996).

[63] N. S. Lawson, A simple heat switch for use at millikelvin temperatures, Cryogenics, 667 (1982).

[64] D.I. Bradley, M. Clovecko, S.N. Fisher, D. Garg, A. Guénault, E. Guise, R.P. Haley, G.R. Pickett, M. Poole, V. Tsepelin, Thermometry in Normal Liquid He-3 Using a Quartz Tuning Fork Viscometer, J. of Low Temp. Phys. 171, 750 (2013); D.I. Bradley, P. Crookston, S.N. Fisher, A. Ganshin, A. Guénault, R.P. Haley, M.J. Jackson, G.R. Pickett, R. Schanen, V. Tsepelin, The damping of a quartz tuning fork in superfluid He-3-B at low temperatures, J. of Low Temp. Phys. 157, 476 (2009).

[65] F. Pobell, *Matter and Methods at Low Temperatures*, Springer (2007).

[66] V. P. Adiga, B. Ilic, R. A. Barton, I. Wilson-Rae, H. G. Craighead, and J. M. Parpia, Approaching intrinsic performance in ultrathin silicon nitride drum resonators, J. Appl. Phys. Vol. 112, 064323 (2012).

[67] D. Cattiaux, S. Kumar, X. Zhou, A. Fefferman and E. Collin, Geometrical Nonlinearity of Circular Plates and Membranes: an Alternative Approach, J. Appl. Phys. Vol. 128, 104501 (2020).

[68] Marc Sansa, Eric Sage, Elizabeth C. Bullard, Marc Gély, Thomas Alava, Eric Colinet, Akshay K. Naik, Luis Guillermo Villanueva, Laurent Duraffourg, Michael L. Roukes, Guillaume Jourdan and Sébastien Hentz, Frequency fluctuations in silicon nanoresonators, Nature Nanotechnology Vol. 11, pp. 552558 (2016).

[69] Olivier Maillet, Dylan Cattiaux, Xin Zhou, Rasul R. Gazizulin, Olivier Bourgeois, Andrew D. Fefferman, and Eddy Collin, Nanomechanical damping via electron-assisted relaxation of two-level systems, arXiv:2009.03804 (2020).

[70] A.D. Fefferman, R.O. Pohl, A.T. Zehnder, and J. M. Parpia, Acoustic properties of amorphous silica between 1 and 500 mK, Phys. Rev. Lett. Vol. 100, 195501 (2008).

[71] A.N. Cleland, *Foundations of Nanomechanics*, Spinger (2003).

[72] M. Tinkham, *Introduction to superconductivity*, Second Edition McGraw-Hill New York (2004).

[73] V.N. Zavaritskii, Investigation of the thermal properties of superconductors. II, J. Exptl. Theoret. Phys. (U.S.S.R.) Vol. 34, 1116-1124 (1958).

[74] J.M. Ziman, *Electrons and Phonons*, Clarendon Press, Oxford (2001).

[75] E.T. Swartz and R.O. Pohl, Thermal boundary resistance, Rev. Mod. of Phys. Vol. 61, 605 (1989).

[76] J.S. Heron, T. Fournier, N. Mingo and O. Bourgeois, Mesoscopic size effects on the thermal conductance of silicon nanowire, Nano Lett. Vol. 9, 1861 (2009); J. S. Heron, T. Fournier, N. Mingo, and O. Bourgeois, Mesoscopic Size Effects on the Thermal Conductance of Silicon Nanowire, Nano Lett. Vol. 10, 2288 (2010).

[77] H.B.G. Casimir, Note on the conduction of heat in crystals, Physica Vol. 5, 495 (1938).

[78] T.A. Puurtinen, and I.J.Maasilta, Radial phononic thermal conductance in thin membranes in the Casimir limit: Design guidelines for devices, AIP Advances Vol. 4, 124503 (2014).

[79] E. Nazaretski, V. O. Kostroun, S. Dimov, R. O. Pohl and J. M. Parpia, Heat Inputs to Sub-mK Temperature Cryostats and Experiments from γ-Radiation and Cosmic Ray Muons, J. of Low Temp. Phys. Vol. 137, pp. 609623 (2004).

[80] O. Usenko, A. Vinante, G. Wijts, and T. H. Oosterkamp, A superconducting quantum interference device based read-out of a subattonewton force sensor operating at millikelvin temperatures, Appl. Phys. Lett. Vol. 98, 133105 (2011).

[81] M. C. Cross, Ron Lifshitz, Elastic wave transmission at an abrupt junction in a thin plate with application to heat transport and vibrations in mesoscopic systems, Phys. Rev. B, Vol. 64, 085324 (2001).

[82] M.P. Blencowe, Quantum energy flow in mesoscopic dielectric structures, Phys. Rev. B Vol. 59, 4992 (1999).

[83] K. Schwab, E. Henriksen, J. Worlock, and M.L. Roukes, Measurement of the quantum of thermal conductance, Nature (London) Vol. 404, pp. 974 (2000).

[84] Adib Tavakoli, Kunal Lulla, Thierry Crozes, Natalio Mingo, Eddy Collin, and Olivier Bourgeois, Heat conduction measurements in ballistic 1D phonon waveguides indicate breakdown of the thermal conductance quantization, Nature Comm. Vol. 9, 4287 (2018).

[85] M.L. Roukes, M.R. Freeman, R.S. Germain, R.C. Richardson, M.B. Ketchen, Hot Electrons and Energy Transport in Metals at Millikelvin Temperatures, Phys. Rev. Lett. Vol. 55, 422 (1985).

[86] M. Meschke,J. P. Pekola, F. Gay, R. E. Rapp, and H. Godfrin, Electron Thermalization in Metallic Islands Probed by Coulomb Blockade Thermometry, J. of Low Temp. Phys. Vol. 134, 1119 (2004).
